# Supplementary material for: Multiple domains of scaffold Tudor protein play nonredundant roles in Drosophila germline
Source: Life Sci Alliance. 2025 Jul 14;8(10):e202503304. doi: 10.26508/lsa.202503304 (PMC12261137; doi:10.26508/lsa.202503304)
Supplement: Supplementary file 1 [file LSA-2025-03304_TableS1.doc]

**Table S1. Statistical analysis of co-occurrence of Tudor protein in Vasa granules in germ plasm of early embryos.**

|  | % granules (Mean ± s.e.m.) | number of embryos/ number of granules | t-test | *P* value |
| --- | --- | --- | --- | --- |
| wild-type | 62.88 ± 4.20 | 8/1291 |  |  |
| *tud*^dom2^ | 50.88 ± 7.24 | 8/849 | 1.43 | 0.17 |
| *tud*^dom4^ | 54.11 ± 7.40 | 9/1129 | 1.00 | 0.34 |
| *tud*^dom5^ | 54.00 ± 8.95 | 4/251 | 1.04 | 0.32 |

This table shows the percentage of granules with both Tud and Vas out of all granules which contain Vas. Unpaired two-tailed *t*-test and *P* values indicate no statistical difference between each of the mutants and *wt* control.
