# Supplementary material for: Multiple domains of scaffold Tudor protein play nonredundant roles in Drosophila germline
Source: Life Sci Alliance. 2025 Jul 14;8(10):e202503304. doi: 10.26508/lsa.202503304 (PMC12261137; doi:10.26508/lsa.202503304)
Supplement: Supplementary file 2 [file LSA-2025-03304_TableS2.doc]

**Table S2. Sequences of genomic sites in *tud* locus targeted by gRNAs used to introduce deletions in Tud domains with CRISPR/Cas9 methodology.**

| Targeted Tud domain | gRNA-targeting site at the beginning of Tud domain/protospacer adjacent motif (PAM) is highlighted | gRNA-targeting site at the end of Tud domain/PAM is highlighted |
| --- | --- | --- |
| **2** | 5′-GAAGCTCAAGGCACTCGCGCAGG-3′ | 5′-TCAAATACCCCCAGAACTGTTGG-3′ |
| **3** | 5′-GCGCCCAAGATCAACTGATCGGG-3′ | 5′-CCAGATGCTTCTCAGCAATATGG-3′ |
| **4** | 5′-ACACCAGCCACTTCAATTAAAGG-3′ | 5′-CTCGGAGGATATTTGGCAATTGG-3′ |
| **5** | 5′-TACCAGGCGCCACTTCGGTAAGG-3′ | 5′-AACAAATTTAGCATCGTCATAGG-3′ |
| **6** | 5′-CTCTTTGAAAAAATTTGATGTGG-3′ | 5′-GCGTGACGATATCAAGGCATTGG-3′  5′-AATAAAGCGTGACGATATCAAGG-3′ |

In addition to targeted sequences for Tud domain 2-5, the corresponding sequences for Tud domain 6 are shown. We could not generate a deletion mutant for this domain, even though two gRNAs targeting the domain’s end were used with common gRNA targeting its beginning in different experiments,
